# Real-time TMS-EEG for brain state-controlled research and precision treatment: a narrative review and guide

**DOI:** 10.1088/1741-2552/ad8a8e

**Published:** 2024-11-01

**Authors:** Miles Wischnewski, Sina Shirinpour, Ivan Alekseichuk, Maria I Lapid, Ziad Nahas, Kelvin O Lim, Paul E Croarkin, Alexander Opitz

**Affiliations:** 1 Department of Psychology, Experimental Psychology, University of Groningen, Groningen, The Netherlands; 2 Department of Biomedical Engineering, University of Minnesota, Minneapolis, MN, United States of America; 3 Department of Psychiatry and Behavioral Sciences, Northwestern University, Chicago, IL, United States of America; 4 Department of Psychiatry & Psychology, Mayo Clinic, Rochester, MN, United States of America; 5 Department of Psychiatry and Behavioral Sciences, University of Minnesota, Minneapolis, MN, United States of America

**Keywords:** closed-loop neuromodulation, transcranial magnetic stimulation, electroencephalography, precision treatment, brain states

## Abstract

Transcranial magnetic stimulation (TMS) modulates neuronal activity, but the efficacy of an open-loop approach is limited due to the brain state’s dynamic nature. Real-time integration with electroencephalography (EEG) increases experimental reliability and offers personalized neuromodulation therapy by using immediate brain states as biomarkers. Here, we review brain state-controlled TMS-EEG studies since the first publication several years ago. A summary of experiments on the sensorimotor mu rhythm (8–13 Hz) shows increased cortical excitability due to TMS pulse at the trough and decreased excitability at the peak of the oscillation. Pre-TMS pulse mu power also affects excitability. Further, there is emerging evidence that the oscillation phase in theta and beta frequency bands modulates neural excitability. Here, we provide a guide for real-time TMS-EEG application and discuss experimental and technical considerations. We consider the effects of hardware choice, signal quality, spatial and temporal filtering, and neural characteristics of the targeted brain oscillation. Finally, we speculate on how closed-loop TMS-EEG potentially could improve the treatment of neurological and mental disorders such as depression, Alzheimer’s, Parkinson’s, schizophrenia, and stroke.

## 1. Introduction

Transcranial magnetic stimulation (TMS) is a non-invasive neuromodulation method which can induce supra- threshold changes in neural membrane potentials in relatively focal neocortical areas [1]. These changes enable probing brain function and delivering neuroplastic interventions [1–3]. TMS is used to interfere with or alter neural activity. Repetitive TMS (rTMS) protocols have been approved by the Food and Drug Administration (FDA) for the treatment of major depressive disorder, obsessive-compulsive disorder, and smoking cessation [4]. Furthermore, rTMS paradigms are studied for the remedy of several other disorders, including stroke [5, 6], Parkinson’s disease [7–9], Alzheimer’s disease [10], schizophrenia [11], and more. A recent umbrella review on rTMS efficacy in major depressive disorder suggested that response rates vary between 62% and 78% [12]. For obsessive-compulsive disorder, response rates have been estimated to be around 50% [13]. While this shows that TMS is a promising method to improve the lives of people with brain-related disorders, a significant number of individuals do not respond to TMS. By advancing the technology, the hope is to improve the response rates.

Importantly, the brain is a dynamic system and brain state impacts TMS effects in a manner that is incompletely understood and underestimated in current research protocols [14, 15]. Most protocols that operate at standard stimulation frequencies do not interact with the dynamic nature of the brain. Neuronal populations engaged in an activity will be affected differently by TMS compared to neuronal populations at a state of rest. Further, repeated causal perturbation, via rTMS, causes a build-up of effects that can lead to plastic changes in cortical activation [2, 3]. Given the state dependency, cumulative effects of rTMS will also rely on ongoing brain activation [14]. It follows that TMS alone, without guidance of real-time spatiotemporal information on brain activity, is suboptimal for making causal conclusions and inducing lasting therapeutic effects. Real-time approaches that combine a readout of cortical activation to guide TMS delivery hold the promise of addressing these current limitations.

Electroencephalography (EEG) non-invasively measures current brain state with excellent temporal and reasonable spatial information. EEG has a millisecond temporal resolution and a spatial resolution of several centimeters. The use of center-surround referencing (e.g. Hjorth or Laplacian) can improve spatial resolution, although the ill-posted inverse problem (the possibility of multiple solutions to the surface distribution) does not allow for precise localization of cortical regions of EEG origin. EEG records a proxy of local field potentials (LFPs), which reflect the fluctuations of extracellular activity. These extracellular events are tightly linked to the timing of neural firing, referred to as spike field coupling [16]. Decades of evidence suggest that the preferential occurrence of spiking at particular phases of ongoing LFPs is related to behavioral performance and synaptic plasticity [17, 18]. For example, hippocampal spikes synchronized to distinct phases of the theta rhythm relate to unique spatial memories [19]. This phenomenon has since been replicated in cortical brain regions [17, 20–22]. Besides memory, neural phase coupling contributes to sustain attention, and insufficient or abnormal attention could be related to decreased spike field coupling [23]. Other mechanisms that rely on increased phase-specificity of neural firing to ongoing oscillatory rhythms include working memory, sensorimotor integration, and perceptual awareness [24]. Consequently, abnormalities in spike-field coupling have been found in several brain disorders including major depressive disorder, schizophrenia, Parkinson’s disease, and Alzheimer’s disease [24–28]. From this follows the rationale four coupling TMS, which can induce neural spiking, with measures of local brain oscillations, such as EEG. As such, TMS-induced action potentials can be synchronized to a particular phase of a neural oscillation, driving the mechanisms of spike field coupling. While phase-dependency can be investigated with a post hoc sorting approach [29], using real-time algorithms has important advantages. Post hoc approaches rely on averaging of trials or correlation analyzes, whereas real-time stimulation allows for investigating a causal relationship between oscillations and behavior by directly modulating them and investigating the subsequent effects on task performance. Furthermore, in clinical settings where TMS is used as an interventional rather than an investigational tool, an real-time method is required for personalization of the treatment.

Recent advances in EEG acquisition and signal processing allow for the integration of TMS and EEG [30]. Integrated TMS-EEG facilitates brain state-controlled perturbation of cortical brain regions [31–33]. Using brain states as neuromarkers reduces inter- and intra-individual variability and opens the gates towards personalized TMS therapies [34]. The clinical effect size of therapeutic TMS is limited in many neuropsychiatric disorders such as major depressive disorder (MDD) [35]. Real-time TMS-EEG is one critical aspect of broader efforts to understand and advance therapeutic applications of TMS. The aim is not to replace, but rather enhance already successful treatments. Notwithstanding, real-time TMS-EEG has a unique set of advantages, both for clinicians, as well as for patients. First, TMS-EEG is non-invasive with minimal side effects. Therefore, a broad spectrum of symptom severity can be treated. Whereas invasive neuromodulation has proven to be reasonably effective, it is reserved for patients with high symptom severity [36, 37]. TMS-EEG, on the other hand, can be applied at the early onset of disorders or even in a preventative way. Second, real-time TMS-EEG allows for personalized precision treatment. TMS has a good spatial resolution and EEG has an excellent temporal resolution, meaning that specific brain networks can be targeted at highly dynamic states. Consequently, TMS is more precise than other non-invasive neuromodulation treatments such as transcranial direct current stimulation, or electroconvulsive therapy. Third, real-time EEG is cost-effective and scalable for clinical practice. Although the initial costs of purchasing TMS and EEG equipment that allows for real-time processing are considerable (up to 200 000 US Dollars), running costs are very low. Costs per interventional session are considerably lower for TMS therapy compared to electroconvulsive therapy. Although TMS is FDA approved, real-time approaches with a personalized approach may decrease the number of sessions in a treatment course yielding substantial financial and pragmatic benefits for patients and payers.

In this article we discuss the recent advances in real-time TMS-EEG. We argue for the importance of neural oscillations as a proxy for brain state in neurological and mental disorders. Next, we summarize real-time TMS-EEG findings that demonstrated the importance of neural oscillation state in cortical excitability. This is followed by a discussion on the potential of real-time TMS as a therapeutic method for the treatment of major depressive disorder, Alzheimer’s disease, Parkinson’s disease, schizophrenia, and recovery after stroke. We will also provide a guide for successful implementation of real-time TMS-EEG. Finally, we provide an outlook for the long-term future of adaptive closed-loop technology and how it potentially could be implemented in clinical research. Note that first clinical trials using closed-loop technology are ongoing and that the state-of-the-art is rapidly evolving.

## 2. Neural oscillations and oscillopathies

Neuronal firing and oscillations reflect two codependent aspects of brain activity and the coupling between these are crucial for network communication [38]. Several lines of evidence from both preclinical and human studies suggest that neural oscillations bias the probability of spiking. Furthermore, strengthening endogenous oscillations by means of exogenous transcranial alternating current stimulation (tACS) increases spiking-timing bias [39–43]. On the macroscopic scale oscillations can be measured non-invasively using EEG. Two prominent measures, power and phase, reflect inhibitory/excitatory network balance at a given point in time and influence subsequent perception and behavior [44]. Various experimental studies have demonstrated that phase and power of oscillations also translate to sensory, motor, and cognitive functioning [31, 32, 45–49].

If human perception and action depends on oscillation dynamics, it is not surprising that pathological oscillations can be indicative of neurological or psychiatric disorders. Various psychiatric disorders are characterized by abnormalities in brain rhythms, referred to as oscillopathies. Neurological disorders including Parkinson’s disease, essential tremor, and dystonia are associated with excessive rhythmic activity and spike-field coupling [26, 50, 51], specifically, increased motor cortical beta-gamma coupling with motor symptoms [52]. Mood disorders, such as MDD also have pathological oscillatory changes [53, 54]. Affective symptoms relate to a prefrontal asymmetry, with increased alpha power in the left and decreased alpha power in the right hemisphere [28, 55, 56]. Additionally, emotion regulation is associated with abnormal theta activation [28]. Given these oscillatory markers, precision targeting of specific oscillatory features with TMS is likely to improve therapeutic efficacy. TMS is particularly useful for real-time targeting of oscillations because TMS pulses are brief (<1 ms) and, therefore, offer excellent temporal resolution to target particular features of an oscillation (e.g. phase). Furthermore, artifacts in the EEG are limited to a few milliseconds, allowing for the simultaneous use of both methods.

## 3. Real-time TMS-EEG predictive methods

Over the past years several algorithms have been developed to target different oscillation states in real time. Targeting the oscillation phase is thought to have particular promise. Detection of instantaneous oscillation phase is challenging because of technical limitations such as hardware and software delays, and phase distortion due to causal band-pass filtering [30]. Multiple methods have been suggested for the estimation of phase in real-time. The majority of these methods rely on forward prediction of the signal to resolve the technical challenges mentioned above. Current methods for phase-specific TMS-EEG fall into three categories: (1) forward prediction of EEG signals in the time domain; (2) forward prediction of phase information in the frequency domain; (3) educated forward prediction of predefined EEG biomarkers. Forward prediction of the EEG signal in the time domain relies on using the temporal characteristics of the data window recorded in real-time to project the signal in the future. For example, auto-regressive (AR) models are commonly used for this purpose [31, 57, 58]. In this method, an AR model of fixed order is fit to the last window of acquired data samples and then future data samples are predicted one by one by feeding the available data in the AR model. The phase of predicted signals are then calculated using Hilbert transform. Similarly, forward prediction of the EEG phase in the frequency domain utilizes the features in the frequency domain for future prediction of the signal, e.g. fast Fourier transform [59], or wavelet transform [60]. In these methods, the frequency components of the last acquired data window is calculated and the periodic frequency components are extended to predict future signals. The educated forward prediction methods rely on training the neuromarker identification and prediction on pre-recorded EEG data before using the algorithm in real-time. In this category, the informative data patterns are extracted once from the prerecorded training data, instead of doing so for each data window acquired online. For example, educated temporal prediction (ETP) algorithm [61] uses a short (∼3 min) EEG from the individual in order to personalize the prediction of a specific phase, such as alpha peak. In this method, the typical distance of the signal peaks at the band of interest is calculated from training data and during the real-time prediction, this distance is projected forward from the last detected peak in the acquired data window. Only the desired phase is predicted rather than the whole signal. Furthermore, other methods have been proposed that do not rely on forward prediction. For instance, machine learning has been used to estimate the phase directly from unfiltered EEG signals [62]. Also, ad hoc methods have been utilized for phase-locked stimulation, such as [63], which incorporated thresholding to detect the peak and trough of the signal. Applying machine learning could also be used to further optimize training algorithms for closed-loop applications. Additionally, it allows for unsupervised network and connectivity analyzes that could guide stimulation parameters [64–66]. For instance, machine learning approaches may unearth treatment predictions for brain-related disorders [64, 67]. It is worth noting that in the closed-loop TMS-EEG framework, the EEG data is a source of independent variables that trigger stimulation. One could also use EEG as a source of dependent variables, for example, by quantified TMS-evoked potentials or TEPs [68]. To date, this approach is rarely used and remains outside the scope of the present review.

## 4. Real-time assessment of cortical excitability

**Figure 1.**
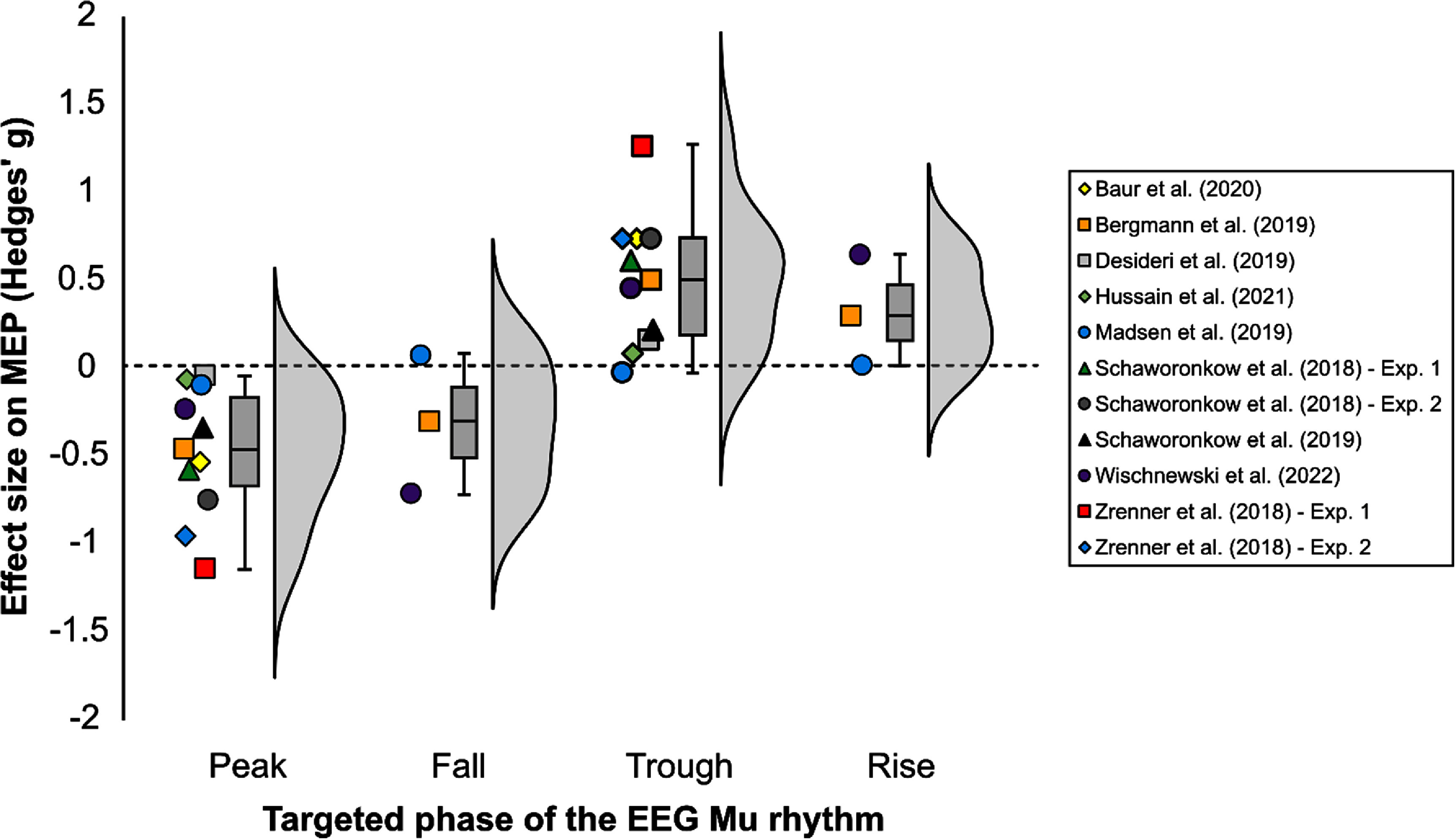
Summary of studies on closed-loop TMS-EEG over primary motor cortex targeting phases of the sensorimotor mu rhythm. Effect sizes represent the effect of the target phase on motor-evoked potential amplitude, normalized to the grand average and expressed in Hedges’ g. On the *x*-axis the phase of the mu rhythm is represented that was targeted by TMS. Note the inverse relationship between the mu phase and MEP amplitude, with the largest responses at the trough and the smallest responses at the peak phase.

The motor cortex is often the focus of studies examining the effects of oscillation states. With presence of monosynaptic connections to peripheral muscles, TMS of the primary motor cortex allows for objectively measurable muscle responses, referred to as motor-evoked potentials (MEPs) [1]. This provides the most direct non-invasive assessment of cortical excitability using TMS. As such, effects of real-time TMS-EEG on MEPs have been explored related to motor cortical oscillatory rhythms [31, 32]. To date, the most investigated rhythm is the sensorimotor mu rhythm (8–13 Hz). As visual alpha oscillations, the mu rhythm has a clear peak during a resting-state, and consequently an excellent signal-to-noise ratio (SNR) [69]. We summarized 10 studies investigating sensorimotor excitability in relation to mu oscillation phase using real-time TMS-EEG (table 1) [31, 32, 60, 70–76]. Despite the use of different real-time algorithms, spatial filters, and probing intensities, the summary of these results shows a clear phase-dependency of MEPs relative to a TMS pulse (figure 1). Cortical excitability is significantly increased at the mu trough and rising phase. Decreased responses can be observed at the mu peak and falling phase. A question that may arise is why the trough/rising phase specifically leads to increased cortical excitability. EEG signals recorded in a Laplacian montage with C3 in the center mostly originate from the gyral crown of the pre and post-central gyrus [1, 77]. A negative deflection (the trough) corresponds to an inward dipole direction, corresponding to the natural current direction of depolarized neurons in the gyral crown. As such, the trough of the mu rhythm could reflect the most excitable state of these neurons [29]. It should be noted that the neurons in the pre-and post-central gyral crowns are only indirectly responsible for muscle output through monosynaptic or disynaptic connections with upper motor neurons [1]. The upper motor neurons with direct connections to the muscle are located in the posterior wall of the precentral gyrus, to which EEG is much less sensitive. This implies that which phase is the most optimal for stimulation largely depends, in part, on neural orientation. However, other factors, such as functional connectivity and the frequency band of the oscillation, should be considered as well [32]. Thus, while the trough/rising phase leads to increased cortical excitability, it could be different for different frequencies and brain regions.

**Table 1.** Overview of studies testing role of mu rhythm phase in corticospinal excitability using real-time phase-specific TMS-EEG. Closed-loop (CL) methods were based on auto-regression (AR), wavelets, or educated temporal prediction (ETP). All papers used spatial filtering centered around the C3 electrode, such as 4-eletrode Hjorth, 8-electrode Laplacian, or spectro-spatial decomposition (SSD). The TMS pulse intensity was expressed in % motor threshold (MT) or as intensity necessary to induce 1 mV motor evoked potential (MEP). The phase specific effects (peak/fall/trough/rise) given here are recalculated in Hedges’ g.

Study	CL method	N	Target	Spatial filter	Intensity	*g* peak	*g* fall	*g* trough	*g* rise
Baur *et al* [70]	AR	12	C3	Hjorth 4	1 mV	−0.53		0.74	
Bergman *et al* [71]	AR	23	C3	Hjorth 4	1 mV	−0.46	−0.30	0.50	0.30
Desideri *et al* [72]	AR	12	C3	Hjorth 4	110% MT	−0.04		0.16	
Hussain *et al* [73]	AR	17	C3	Hjorth 4	115% MT	−0.06		0.08	
Suresh and Hussain [74]^a^	AR	23	C3	Hjorth 4	120% MT	↓		↑	
Madsen *et al* [60]	Wavelet	14	C3	Hjorth 4	1 mV	−0.10	0.07	−0.03	0.02
Schaworonkow *et al* [75]	AR	14	C3	Hjorth 4	112% MT	−0.58		0.61	
Schaworonkow *et al* [75]	AR	14	C3	SSD filter	112% MT	−0.75		0.74	
Schaworonkow *et al* [76]	AR	15	C3	SSD filter	Variable	−0.35		0.22	
Wischnewski *et al* [32]	ETP	20	C3	Laplace 8	120% MT	−0.23	−0.71	0.45	0.64
Zrenner *et al* [31]	AR	12	C3	Hjorth 4	1 mV	−1.14		1.26	
Zrenner *et al* [31]	AR	11	C3	Hjorth 4	1 mV	−0.95		0.74	

^a^ Average MEP could not be extracted from this paper, but general trends (larger MEP for trough compared to peak) were similar to other studies.

Given the generally smaller amplitude, SNR for beta oscillations (13–30 Hz) tends to be lower. Furthermore, extra care should be taken during analysis to minimize the contribution of mu harmonics [78]. Recently, studies demonstrated that beta phases can be targeted reliably in real-time [32, 79]. In our study we showed a phase-dependent response for beta phases that is opposite compared to mu, with largest MEP amplitudes at the falling phase and smallest MEP amplitudes at the rising phase [32].

Besides phase, several groups have used TMS-EEG triggered in real-time by oscillatory power. Results for mu power-triggered TMS show a positive relationship between power and cortical excitability [74, 80–82]. However, these effects are subtle and are sensitive to spatial filtering, emphasizing the importance of extracting the optimal locus of the signal. Several studies also have looked at the effects of online beta power on MEPs, however no significant association has been observed to date [32, 82].

## 5. A guide for successful implementation of real-time TMS-EEG

**Figure 2.**
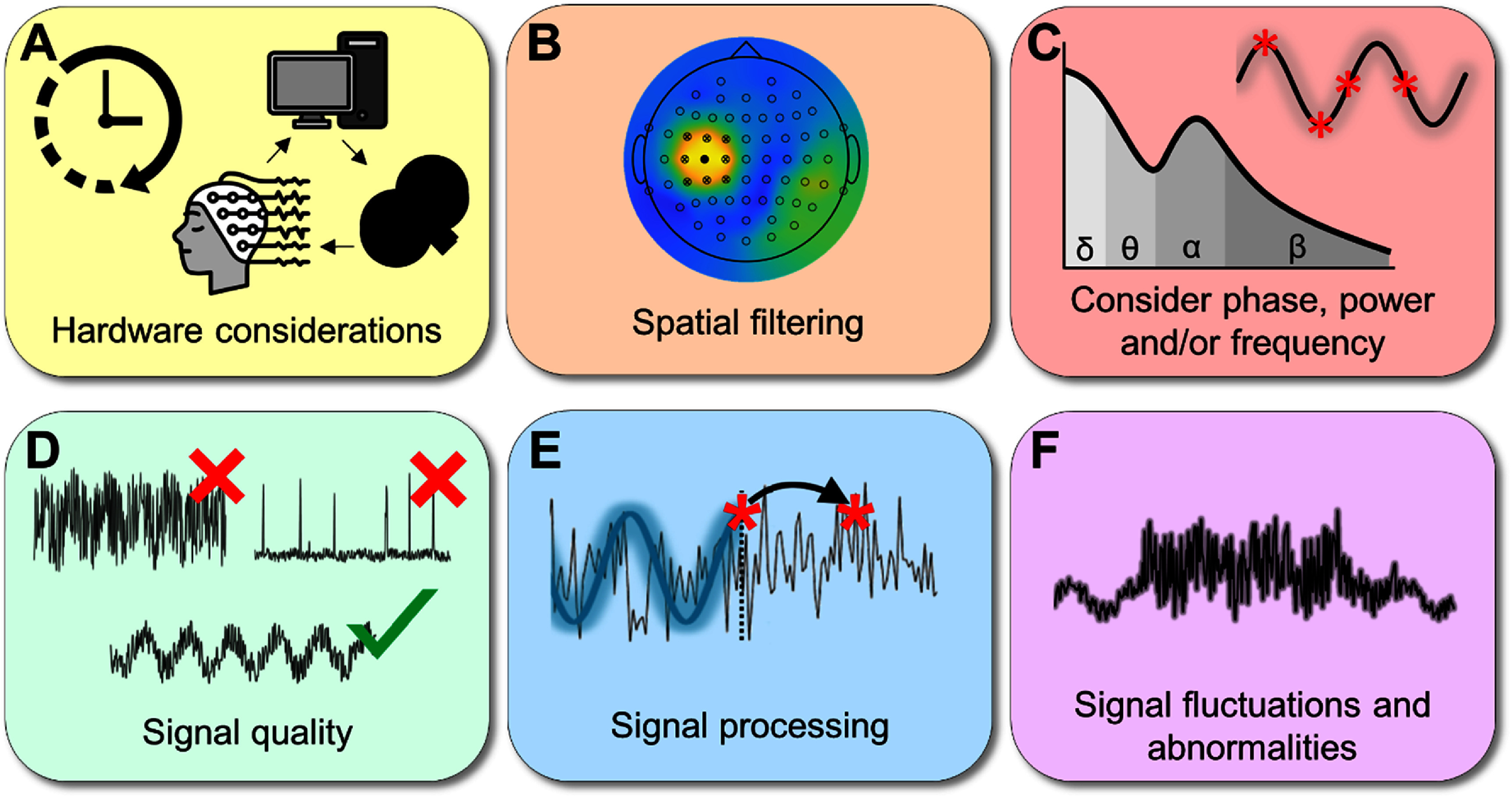
Six factors that are crucial for successful closed-loop experiments, namely, (A) the hardware processing and transmission delays, (B) extraction of the signal of interest using frequency and spatial filtering, (C) defining the neuromarker of interest in terms of frequency band, power, and phase of the brain rhythm, (D) requirements for signal quality for the algorithm of choice, (E) the algorithm for closed-loop processing, (F) strategy for operations in the presence of strong extracephalic or technical noise.

The previously mentioned algorithms are, in principle, fast enough to perform real-time triggering on brain signals up to and including the fast beta-rhythm (13–30 Hz) [61]. To date, all algorithms stream or downsample EEG data at 500 Hz–1000 Hz sampling rate and process the data sample-by-sample. Such sampling rates allow characterization of quasi-sinusoidal brain oscillations in a typical EEG range of under 40 Hz. Thus, all computational processes should be performed per each data sample faster than the next sample arrive (<2 ms for 500 Hz sampling or <1 ms for 1000 Hz sampling). Most real-time system delays come from the hardware (figure 2(A)), which can be around 10 milliseconds (note that systems are specialized for fast operation, such as Turbolink from BrainProducts). TMS-EEG compatible equipment is essential for real-time approaches with TMS to provide fast data streaming and processing. Flat electrodes are preferred to minimize coil-brain distance and the equipment should be able to avoid TMS-related saturation (high dynamic range amplifier). Currently established methods use forward prediction to account for the delays. Since the accuracy of such predictions decreases drastically with projection distance, the period between current state and projected state should be as short as possible [83]. Furthermore, it is crucial to consider different TMS devices and the main parameters of stimulation. For instance, paired-pulse paradigms, such as short-intracortical inhibition, require the ability to provide consecutive pulses within a few milliseconds [84]. In a similar vein, parameters such as different pulse widths, pulse forms (monophasic, biphasic), and pulse directions can shape TMS-induced cortical responses (for review articles, see [1, 85]). Currently, the majority of studies applied closed-loop TMS at the motor cortex (but see also [34, 86]) with biphasic posterior-anterior pulses and a coil angle of 45 degrees relative to the posterior-anterior axis (e.g. [31, 32, 71]). This means that the current direction is most aligned with the somatodendritic axis in the primary motor cortex [1]. Previous studies have shown that changes in coil orientation relate to differences in cortical excitability and expression of TMS-induced direct and indirect waves [87]. Likely these parameters will strongly affect the results that can be obtained from closed-loop TMS-EEG. However, to date, the effects of different TMS parameters have not yet systematically been studied in combination with real-time EEG, and further research is required.

One paramount factor that determines the success of real-time approaches is the quality of the signal (figure 2(B)). Higher SNR correlates to higher accuracy of targeting desired states, such as oscillation phases [61]. The SNR will depend on a variety of factors. Thorough preparation is required to ensure low impedances between electrode and skin. Also, the use of gel-based equipment instead of dry caps ensures good signal quality for prolonged sessions. Besides equipment, SNR also depends on the region and rhythm of interest. For instance, occipital alpha oscillations naturally have a high SNR, particularly when in a closed-eyes condition [88]. Conversely, beta oscillations are significantly lower in amplitude [61], and for lower frequencies, like theta, there is a relatively higher contribution of non-periodic activity [89]. Despite these biological limitations, successful targeting of different theta phases has been demonstrated [86]. Some algorithms use training data to inform subsequent recordings [61]. In this way algorithm parameters can be based on a large amount of data and only need to be adjusted in real-time processing, which reduces processing speed and improves accuracy. Crucially, the data used for training should be equivalent to real-time data. For instance, when real-time TMS-EEG is performed on a resting state with open eyes, the training data should be collected also at rest with open eyes [61]. Finally, non-neurogenic artifacts, like muscle and eye movements, also affect SNR. This could be of particular concern when working with neurological patients (e.g. Parkinson’s disease). This has prompted the development of real-time artifact removal [90, 91].

Defining the region of interest (e.g. primary motor cortex, dorsolateral prefrontal cortex, etc.) it is crucial to optimize spatial filtering (figure 2(C)). Although spatial resolution with EEG is limited, it can be optimized using center-surround montages, such as Laplacian or Hjorth spatial filtering. In other words, an electrode of interest is surrounded by reference electrodes, meaning that the averaged signal of surrounding electrodes is subtracted from the center electrode. Typically, an electrode above the region of interest, such as C3 for left primary motor cortex and F3 for left dorsolateral prefrontal cortex is selected for the main read out. However, extra care should be taken in the case of cortical lesions, which distort spatial EEG maps, resulting in illusory smoothness [92, 93]. Using high-density caps (⩾64 channels) mitigates this problem to a certain extent [93], but does not eliminate the issue. Alternatively, spatial filtering can be applied to project the signal to the source space, in contrast to the sensor space [60, 75, 76]. Regardless of filtering method, lesions will affect TMS-induced electric fields [94, 95], meaning that coil position needs to be adjusted. As such, for encephalopathies spatial filtering requires additional steps and adjustments compared to healthy volunteers and structural and functional imaging can be beneficial.

Within the time domain, temporal filtering and window length are further key components in determining the accuracy of a real-time algorithm (figure 2(D)). Concerning temporal filtering, used for extracting certain oscillation frequency band, one issue is the edge artifact, which is a distortion that occurs at the beginning or end of an EEG segment. This causes significant phase shifts [30, 61]. As such, forward predictions should be made based on the undistorted signal. Filters also vary by the amount edge artifact, sharpness, and time costs, which should be accounted for during the algorithm development and testing. The important computational step of closed-loop TMS-EEG is a real-time extraction of the brain oscillation of interest. This is most commonly done with a digital filter. Selecting an appropriate filter is crucial as all of them come with compromises. For example, causal filters are faster but can introduce phase distortions, while noncausal filters require extra processing time. We refer readers to the specialized reviews for more details [96, 97]. Example choices for implemented real-time phase detection methods include low-order forward–reverse FIR filter and non-causal sinc (brick wall) filter. The FIR filters induce no distortions in the passband but are more time costly and can lack the required sharpness for very narrow brain rhythms (thus, picking up information from surrounding rhythms). The brick wall filter is fast and sharp but induces ripples in the time domain [61]. The optimal choice depends on the specific scenario, and the filter of choice should be re-validated for every specific brain area, brain state, and brain rhythm. As discussed in section *‘Real-time TMS predictive methods’*, there are several ways to perform a forward prediction, including autoregression, peak-to-peak estimation, and frequency-dependent methods. However, what all methods have in common is that the prediction is based on the real-time analysis of a short segment of EEG. The window-length of this segment depends on endogenous oscillation of interest. A segment should contain an appropriate amount of oscillation cycles (⩾5) to accurately capture ongoing trends. As such, window lengths of slower frequencies (e.g. theta) should be longer than for faster frequencies (e.g. beta). Given the dynamic nature of cortical oscillations, extending window lengths too much is also undesirable as some data within the segment is no longer informative of the signal in the future, which reduces prediction accuracy. Furthermore, longer segments with more data can add to the computation burden. When dynamics of an oscillation are well known (e.g. the sensorimotor mu rhythm) fixed temporal windows can be used, but for more variable signals (e.g. frontal theta rhythm) optimization approaches for determining window length are recommended.

Besides technical and methodological aspects of a closed-loop system, it is crucial to formulate specific hypotheses (figure 2(E)). Specifically, identify (I) the behavior/cognition/symptoms that should be targeted; (II) the neuroanatomic or neurocircuit that needs to be targeted; (III) reliable electrophysiological neuromarkers (phase, power, frequency) that relate to this behavior/cognition/symptom; and (IV) whether these neuromarkers relate to performance improvements or detriments. Additionally, it is important to monitor broader cognitive states like attention and arousal, as well as general fluctuations and abnormalities in the signal (figure 2(F)). Intake of substances or medication shortly before or during the experiment can lead to signal changes over time, which can hamper closed-loop accuracy. Furthermore, any other stimuli, such as music or video, can drastically alter cortical oscillations. When using training-based algorithms [32, 61], fluctuations of the signal over time will make the training dataset less accurate over time. Therefore, we suggest updating the training set after one hour, as conditions may have changed.

Within an experimental setup it is also important to consider potential control conditions. First, a control condition to demonstrate the phase specificity. A target phase (e.g. peak) should be compared to a control phase (e.g. trough or random). Second, a frequency control demonstrating that observed effects only occur in a target frequency. Third, a location control. Absence of effects should be shown when stimulation location and locus of phase/frequency are not matched. Controlling for all these factors is often impractical given restrictions in time and resources, which is why control conditions should be designed to strengthen the research questions and hypotheses.

## 6. Real-time TMS in clinical settings

The approach of real-time TMS-EEG is of particular interest in disorders that display abnormalities in oscillatory activity. Such abnormalities have been extensively studied in Parkinson’s disease, stroke, Alzheimer disease, major depressive disorder, and schizophrenia. Specifically, Parkinson’s disease is marked by increased beta synchronization in the cortico-basal ganglia motor loop [98]. Memory deficiencies in Alzheimer’s disease are associated with abnormalities in theta and gamma oscillations [27]. Stroke-induced unilateral damage to the brain is associated with an interhemispheric imbalance in alpha and beta oscillations [99, 100]. Frontal alpha oscillation abnormalities have been associated with affective symptoms in depression [101], while theta oscillations may relate to cognitive symptoms [102]. In schizophrenia a lack of phase-consistency can be observed in a variety of oscillations which are linked to positive and negative symptoms [103, 104]. While pathological oscillations are not exclusive to these disorders, given the extensive prior research on their oscillatory characteristics closed-loop TMS-EEG could be particularly interesting. Currently, only a few studies have attempted closed-loop TMS-EEG in a clinical setting. In the section below, we will discuss how real-time TMS-EEG can be a prospective precision treatment for various brain conditions. Note that this section is meant to outline potential future implementations rather than to review existing studies. The efficacy of closed-loop TMS-EEG as treatment compared to standard protocols remains an open question.

### 6.1. MDD

MDD was the first psychiatric disorder for which TMS received FDA approval in 2008. Since that time the clinical use of TMS and research in this area has increased exponentially. Unsurprisingly, a large proportion of research articles in the last 15 years that discuss TMS focus on MDD. Despite multiple TMS dosing options for MDD [105], the outcomes and remission rates are not optimal. There are also unanswered questions as to how to adapt TMS for adolescent [106, 107] and late life MDD [108, 109]. Nevertheless, given the significant number of non-responders, optimizing TMS treatment, by real-time approaches, is still warranted. Animal research has demonstrated that condition fear responses are driven by phase-locked responses in the prefrontal cortex theta (4 Hz) [110]. Further, prefrontal phase-specific (rising or falling phase) optogenetic stimulation at 4 Hz was shown to have opposing effects to freezing behavior [111]. Rising phase stimulation led to reduced, whereas falling phase stimulation led to increased conditioned fear responses. Specifics of optimal phase, frequency, and location for stimulation will likely differ between animal models and non-invasive brain stimulation in humans. Nevertheless, phase-specific real-time TMS over the human prefrontal cortex has been shown to alter oscillatory activity [34] and cognitive performance [86]. Phase-specific TMS bursts targeting the theta rhythm led to faster responses in a working memory task, only if the trough of the oscillation was stimulated. Theta-peak and random stimulation did not alter performance [86]. Also, theta-trough, but not theta-peak TMS bursts led to increased theta power evoked by a single TMS pulse, suggesting both physiological and behavioral changes. Further, Faller *et al* [34] presented interim results of an ongoing clinical trial where phase-specific rTMS is applied for six weeks in patients with major depressive disorder. In this study prefrontal TMS is applied to the personalized preferred phase of the alpha rhythm that resulted in largest responses in the anterior cingulate cortex, as determined in an fMRI session. Interim results of 15 patients suggest that an increase in phase entrainment over the course of several weeks. Although the effects of this phase-specific rTMS on depressive symptoms are currently unclear, these preliminary findings suggest that real-time TMS-EEG can be effective in altering abnormal neurophysiology. Besides phase, another possibility would be to target online alpha power. Depression has been associated with a frontal hemispheric asymmetry in alpha power [112, 113] (but see [114] for a critical review). Real-time analysis of this asymmetry and adjusting stimulation parameters to restore the hemispheric balance could be a potential way of treating major depressive disorder [115, 116].

### 6.2. Parkinson’s disease and essential tremor

A key neuromarker for Parkinson’s disease is hyper-synchronicity of beta oscillations in subcortical and cortical regions [98]. One way to reduce hyper-synchronicity related to Parkinson’s disease symptoms is to apply stimulation to desynchronize rhythms at the tremor frequency. Using such an anti-phase stimulation approach with deep-brain stimulation it was shown to reduce tremors by 87% percent [117]; for a review see [118]. Similarly, using non-invasive alternating current stimulation at a phase that was opposite to the tremor rhythm, reduction in symptoms up to 42% were observed [119]. The drawback of deep brain stimulation is that it requires an invasive approach, which is more suited for patients with advanced symptoms. tACS is spatially imprecise and can only modulate cortical activity at a subthreshold level without directly inducing neural spiking. Real-time TMS-EEG overcomes these limitations, as it is both non-invasive, yet induces neural firing in a spatially precise manner. Non-EEG-guided TMS has already been shown to be effective in resetting tremors [7–9]. Five days of rTMS interventions reduced movement dysfunction up to 12 months after treatment [120]. However, the efficacy of TMS is variable and depends on the specific symptoms [8]. Further, it is important to note that, in contrast to deep-brain stimulation, TMS primarily targets cortical areas. As such, core regions implicated in Parkinson’s disease (e.g. basal ganglia) are stimulated indirectly by placing TMS over the motor cortex [7, 8]. Real-time TMS-EEG has the potential to precisely target tremor phases, as well as cortical oscillation phases that reduce hyper-synchronicity. Converging evidence in healthy volunteers has shown decreased MEP amplitudes at the peak of the sensorimotor mu oscillation [31, 32, 71]. Since increased MEP amplitudes are correlated with Parkinson’s disease symptoms [121, 122], peak-mu TMS could be one possible treatment option. However, systematic experimental research is required to find optimal phase and frequency for TMS-EEG to reduce tremor and motor dysfunctions.

### 6.3. Stroke

Repetitive real-time TMS over primary motor cortex has lasting effects on corticospinal excitability, which indicates synaptic plasticity, akin to long-term potentiation. Increased lasting excitability was observed when the trough of the mu oscillation, but not the peak, was targeted [31]. Whereas open-loop rTMS effects are highly variable between and within individuals, early evidence suggests that mu trough-targeted rTMS suffer from such variability to a much lesser extent. With the ability to reliably induce plastic changes real-time TMS-EEG is a potential breakthrough for post-stroke recovery. Previously, it has been shown that increases in MEP amplitudes after rTMS are positively correlated with larger improvements in motor assessments in stroke patients [5, 6]. As such, long-term increase of mu trough-targeted rTMS might play a key role in movement recovery. Note, real-time applications can be further complicated by structural damage, for example selecting a proper way to extract oscillatory signals (discussed in further detail in ‘*A guide for successful real-time TMS-EEG*’). Nevertheless, recently it was shown that phase-specific real-time TMS can be successfully used in the lesioned hemisphere of stroke survivors [123]. This study included patients with both cortical and subcortical lesions. Particularly cortical lesions can significantly alter mu rhythms, as they are thought to be generated in the sensorimotor cortex. Success rates of real-time TMS on motor responses are currently under investigation (e.g. an ongoing clinical trial: clinicaltrials.gov identifier: NCT04968743).

### 6.4. Alzheimer’s disease

Alzheimer’s disease is characterized by abnormalities in both theta and gamma oscillations, which have been associated with decreased cognitive and executive functioning [27]. Whereas gamma oscillations are difficult to target with real-time TMS-EEG due to their low SNR (for details see ‘*A guide for successful real-time TMS-EEG*’), theta oscillation phases can reliably be pinpointed [86]. Generally, theta oscillations are reported to be decreased in Alzheimer’s disease. Furthermore, memory processes in medial and lateral temporal regions show theta phase preferences [124, 125]. For instance, animal studies have demonstrated that entorhinal-to-CA1 input is typically timed to the trough of the theta oscillation [126, 127], whereas input to CA3 occurs at the peak [128]. Accordingly, phase-specific stimulation of CA1 at the trough of the theta rhythm improves memory accuracy in mice [129]. Whereas the hippocampus is inaccessible for TMS in a direct way, stimulation of the lateral temporal cortex can also yield improved memory [124] and may as such be a potential target for real-time TMS-EEG. Furthermore, Alzheimer disease is associated with deficiencies in the prefrontal cortex. Recently, phase-specific TMS-EEG targeting the prefrontal theta rhythms has shown significant improvements in working memory performance when the theta trough was targeted in healthy volunteers [86]. Whether these results translate to patients is an open-ended question, but preliminary findings demonstrate the feasibility of real-time TMS for improving memory.

### 6.5. Schizophrenia

Schizophrenia is another disorder that is associated with pathological oscillations. In particular discontinuity and lack of phase consistency is observed in delta, theta, and gamma oscillations [103, 104]. As a results, oscillatory power shows abnormal fluctuations [130]. Furthermore, in prefrontal cortex excessive theta activity is observed in response to single pulse TMS [131]. Of particular interest is the locking of neural firing to ongoing oscillations. On the one hand, it has been suggested that neural phase synchronization to ongoing oscillations is reduced in visual organization and auditory processing [25]. On the other hand, abnormally increased prefrontal phase-locking has been related to suboptimal neural plasticity [132]. Given that closed-loop TMS-EEG can induce neural spikes in a phase-dependent manner, this methodology can be used to further investigate neural phase-locking in human participants. A clearer understanding of these seemingly contradicting findings is required before closed-loop TMS-EEG can be used therapeutically. Thus, investigating phase-relationships between TMS-induced theta, beta and gamma oscillations could elucidate a path to potential phase-specific treatment. This is of particular interest since current open loop TMS approaches only have shown mixed success in the treatment of schizophrenia symptoms and generally a deeper fundamental understanding is required [11].

### 6.6. Attention-deficit hyperactivity disorder (ADHD)

ADHD has been associated with abnormalities in theta and beta oscillations. Originally, the ratio between theta and beta oscillations at rest was proposed to be indicative of ADHD symptomatology [133]. However, recent studies have refuted this notion [134, 135]. Nevertheless, neurofeedback studies that target theta and/or beta oscillations have shown positive results in ADHD patients [136, 137]. As an underlying mechanism, it has been proposed that neural phase entrainment at theta frequencies is key for selective attention [23]. Currently, standard TMS seems promising for improving some functions in ADHD, but results remain mixed [138]. With the suggested oscillatory abnormalities of ADHD in mind, closed-loop TMS-EEG could be explored. However, before clinical application, more research is required to fully understand the oscillatory correlates of ADHD, as well as those of other disorders mentioned in this section.

## 7. Challenges and future outlook

The potential clinical application of adaptive and personalized real-time TMS-EEG is vast and holds promise for enhancing neuromodulation treatments in various neurological and neuropsychiatric disorders. Despite its potential, this technology is still in its infancy and randomized clinical trials are required to establish if closed-loop TMS-EEG is superior to an open loop approach, and if so, how much more effective it is. Furthermore, there are some challenges that still need to be addressed. For instance, while current algorithms are very successful in targeting oscillatory states with single pulses, it needs to be investigated whether this also holds for patterned rTMS approaches, such as theta burst stimulation. Furthermore, the current generation of TMS-EEG systems implements a state-controlled stimulation, where predefined TMS intervention is delivered towards a predefined target brain state. Prospective adaptive neurostimulation would not only provide brain-state dependent stimulation, but also adapt stimulation parameters to drive neural activity to a desired state. Whereas in current real-time TMS-EEG experiments timing of stimulation is the only factor that is manipulated, adaptive TMS-EEG would also include changes of stimulation location, intensity, and frequency. Besides power and phase alternative outcome measures such as real-time connectivity and network analyzes could be explored [66, 139]. This could include investigation of microstates [140]. Machine learning approaches are a potential candidate to manage the large parameters space of brain states [141]. For disorders with a relatively well-understood neural pathophysiology, such as Parkinson’s disease, machine learning models can be used to classify neural signals as normal or abnormal. For less well-understood disorders, machine learning models can be used to identify onset of symptoms and predict disease severity. Improving technology may not be the biggest challenge, since current real-time and closed-loop systems are already accurate and fast. Rather, the success of adaptive technology will depend on our understanding of the disorder in question. Deciphering biomarkers explaining symptoms of depression, Alzheimer’s disease, schizophrenia, and so on, will be the main objective for the future of adaptive TMS. Further challenges surround the long-term effects of rTMS, whether it be closed-loop or not. Long-term effects of rTMS beyond a few months are not well-studied [142]. Maintenance treatments can be offered, but there are no guidelines for the frequency of such additional sessions [143].

It should also be acknowledged that current closed-loop approaches have technological limitations. For instance, EEG data at higher frequencies (i.e. gamma range) can be heavily confounded by muscle activity [144], and as such, reliably extracting phase and power information in real-time is difficult [32]. Similarly, TMS itself induces artifacts that disrupt ongoing EEG, which creates a challenge for closed-loop repetitive paradigms. Any estimation of phase requires tracking the signal for a duration that captures enough cycles to create an estimate [61]. This window is too short for high-frequency rTMS (10 Hz, 20 Hz). As such, only the pulse of a high-frequency rTMS burst can be reliably estimated. Further, while closed-loop approaches aim to reduce intra- and inter-individual variability, remaining sources of variance should be considered. For instance, while on a group level the motor cortical TMS at the mu trough phase leads to the largest MEPs (figure 1), this is not per se true for all individuals [32]. Other physiological, structural, and cognitive factors should also be considered.

Bridging the gap between the known potential therapeutic benefits of real-time TMS-EEG and translating them into clinical practice for patients with neurologic and neuropsychiatric disorders requires a multi-faceted and patient-centered approach. First, rigorous clinical trials and longitudinal studies with diverse patient populations are needed to validate the efficacy and safety of TMS-EEG interventions for specific neurologic and neuropsychiatric conditions such as Alzheimer’s, Parkinson’s, stroke, schizophrenia, and major depression. Concurrently, efforts to standardize protocols and guidelines should be prioritized to ensure consistency and comparability across studies and clinical settings. Second, integrating AI strategies into TMS-EEG research can generate AI-driven predictive models that can assist clinicians in selecting optimal TMS-EEG parameters and tailoring therapeutic regimens to individual patient profiles. Third, collaborative efforts between researchers, clinicians, AI experts, industry stakeholders, and regulatory bodies are essential to address safety concerns, streamline regulatory approval process, and facilitate the integration of TMS-EEG technology into clinical practice. Lastly, raising awareness among patients and the broader medical community about the potential therapeutic benefits of real-time TMS-EEG can foster acceptance and support for its incorporation into mainstream clinical care, ultimately maximizing its impact on patient outcomes. By synergizing these strategies, we can accelerate the translation of TMS-EEG’s therapeutic potential into clinical practice, ushering in a new era of innovative and personalized care for patients with neurologic and neuropsychiatric disorders, ultimately improving their quality of life.

## Data Availability

This manuscript is a review and does not contain any data. Therefore, no data can be shared. The data cannot be made publicly available upon publication because they are owned by a third party and the terms of use prevent public distribution. The data that support the findings of this study are available upon reasonable request from the authors.
